# Is there a value for probiotic supplements in gestational diabetes mellitus? A randomized clinical trial

**DOI:** 10.1186/s41043-015-0034-9

**Published:** 2015-11-25

**Authors:** Neda Dolatkhah, Majid Hajifaraji, Fatemeh Abbasalizadeh, Naser Aghamohammadzadeh, Yadollah Mehrabi, Mehran Mesgari Abbasi

**Affiliations:** 1 Department of Nutrition Sciences, Shahid Beheshti University of Medical Sciences (SBUMS), International Branch, tehran, iran; 2 Nutrition Society, National Nutrition and Food Technology Research Institute, Faculty of Nutrition and Food Technology, Beheshti University of Medical Sciences, Baran 3, West Arghavan, Farahzadi Blvd., Shahrak Qods, 19395-4741, istanbul, 1981619573 turkey; 3 Department of Obstetrics & Gynecology, Tabriz University of Medical Sciences, tabriz, iran; 4 Section of Endocrinology & Metabolism, Department of Internal Medicine, Tabriz University of Medical Sciences, Tabriz, Iran; 5 Department of Biostatistics & Epidemiology, Shahid Beheshti University of Medical Sciences, Tehran, Iran; 6 Drug Applied Research Center, Tabriz University of Medical Sciences, Tabriz, Iran

**Keywords:** Probiotics, Gestational diabetes mellitus, Nutrition, Randomized clinical trial

## Abstract

**Background:**

Although several studies have found probiotics encouraging in prevention of gestational diabetes mellitus (GDM), the evidence for the use of probiotics in diagnosed GDM is largely limited. The aim of this study was to assess the effect of a probiotic supplement capsule containing four bacterial strains on glucose metabolism indices and weight changes in women with newly diagnosed GDM.

**Methods:**

Sixty-four pregnant women with GDM were enrolled into a double-blind placebo-controlled randomized clinical trial. They were randomly assigned to receive either a probiotic or placebo capsule along with dietary advice for eight consecutive weeks. The trend of weight gain along with glucose metabolism indices was assayed.

**Results:**

During the first 6 weeks of the study, the weight gain trend was similar between the groups. However, in the last 2 weeks of the study, the weight gain in the probiotic group was significantly lower than in the placebo group (*p* < 0.05). Fasting blood sugar (FBS) decreased in both intervention (from 103.7 to 88.4 mg/dl) and control (from 100.9 to 93.6 mg/dl) groups significantly, and the decrease in the probiotic group was significantly higher than in the placebo group (*p* < 0.05). Insulin resistance index in the probiotic group had 6.74 % reduction over the study period (*p* < 0.05). In the placebo group, however, there was an increase in insulin resistance index (6.45 %), but the observed change in insulin resistance was not statistically significant. Insulin sensitivity index was increased in both groups. The post-intervention insulin sensitivity index in the probiotic group was not significantly different from placebo when adjusted for the baseline levels.

**Conclusions:**

The probiotic supplement appeared to affect glucose metabolism and weight gain among pregnant women with GDM. This needs to be confirmed in other settings before a therapeutic value could be approved.

## Background

Gestational diabetes mellitus (GDM) is a condition in which the pregnant woman has high serum glucose levels during the gestation. It is defined as carbohydrate intolerance first diagnosed during pregnancy [1]. It is characterized by maternal insulin resistance and is associated with inflammation through the gestation [2]. GDM is shown to be associated with a range of adverse pregnancy outcomes such as preeclampsia and abnormal delivery. The newborns may also suffer from some health problems as a consequence. GDM rates are increasing both in high-income as well as the low- and middle-income countries as a consequence of increasing rates of overweight and obesity [3]. Both women with GDM and their infants are at increased risk of diabetes mellitus and metabolic dysfunction later in life [4, 5]. It has been confirmed that treatment of GDM improves pregnancy outcomes with significant reductions in the rate of serious perinatal problems such as macrosomia, dystocia and inevitable caesarean section deliveries. Generally, the treatment includes diet with or without medication [6].

Probiotics are known to be the good bacteria usually consumed as capsules or drinks to supplement the bowl bacteria. As narrated by Salmin, currently, the most commonly used definition for probiotics is given by Fuller as “Probiotics are live microbial feed supplements which beneficially affect the host animal by improving its intestinal microbial balance” [7, 8]. They are shown to affect the consumer’s metabolism. The use of probiotics has been investigated on various infectious or noninfectious conditions [9–13]. Probiotics have also been investigated for their effect on type 2 diabetes or prevention of gestational diabetes [14–21]. Although several studies have found probiotics encouraging in prevention of GDM, the evidence for use of probiotics for those with diagnosed GDM is largely limited. The aim of this study was to assess the effect of a probiotic supplement capsule containing four bacterial strains in comparison with placebo on glucose metabolism indices and weight changes in women with newly diagnosed GDM.

## Methods

In a double-blind placebo-controlled randomized clinical trial, 64 subjects with GDM referred to Alzahra University Hospital in Tabriz, Northwest of Iran, were enrolled during the spring and summer months in 2014. The patients were randomly allocated to receive either probiotic supplement or placebo capsules once daily for 8 weeks.

Each probiotic capsule of four bacterial strains (4 biocap > 4 × 10^9^CFU) in standard freeze-dried culture included *Lactobacillus acidophilus* LA-5, *Bifidobacterium* BB-12, *Streptococcus thermophilus* STY-31 and *Lactobacillus delbrueckii bulgaricus* LBY-27 plus dextrose anhydrous filler and magnesium stearate lubricant produced by CHR HANSEN, Denmark, packed and gelatin covered in Tehran Darou drug industries.

The eligible subjects to be enrolled included all nulliparous women with GDM screened during 24–28 weeks of gestation who were referred to the specialty and subspecialty gynaecology or endocrinology clinics of Tabriz University of Medical Sciences.

The inclusion criteria were as follows: Nulliparity; Gestational diabetes between 24 and 28 week (+6 days) of gestation newly diagnosed through screening done by either a gynaecologist or an internal medicine specialist; Age range of 18–45 years; Fasting blood sugar range of 92 to 126 mg/dl early at the diagnosis; Body mass index (BMI) above 18.5 kg/m^2^; No history of type 2 diabetes mellitus; No history of chronic diseases; No smoking and alcohol consumption; Not using probiotic food products during the 2 weeks before intervention; Not using antibiotics during the month before intervention; Lack of acute gastrointestinal problems a month before trial and Not using Glucocorticoids (GCs) and immunosuppressive drugs. The exclusion criteria were the following: Needing to use insulin of other diabetes drugs through the study period; Use of antibiotics through the study period and Use of GCs and immunosuppressive drugs through the study period.

At baseline, the purpose and method of study were described in detail for the patients, and a trained practitioner provided similar diet recommendations for patients in both groups. Written informed consent was obtained from all the patients for being enrolled into this study. Then, during an interview with the participants, a general questionnaire and a dietary recall questionnaire were completed. The general questionnaire was used to collect data on demographic information, weight before pregnancy, physical activity, past medical history, drug history over the past month and use of probiotic food products over the past 2 weeks before the study. Physical activity was only measured at baseline. Subjects were categorized into three groups: those with low physical activity (those with a sedentary life and physical activity limited to chores such as cooking, sewing, working with computer and so on); those with moderate physical activity whose activity is limited to works such as cleaning or taking care of children and other works needing small amounts of movement or bodily movements and those with high physical activity who used to have brisk walking, running, biking and swimming regularly [22]. A 24-h dietary recall questionnaire was completed at sessions of three nonconsecutive days each (two weekdays and one in weekend) once at the baseline, and then after 4 weeks and also at the end of study. To obtain the nutrient intake of participants based on these 3-day food diaries, we used Nutritionist IV software (First Databank, San Bruno, Calif., USA) modified for Iranian foods. Weight, height and blood pressure were measured, and some information about their dietary habits and weight before pregnancy were also taken. Seca 206 wall-mounted stadiometer and Seca 813 digital scale were used to measure weight and height. Body mass index (BMI) was then calculated and categorized according to the world health organization guidelines [23].

After taking blood samples and randomly allocating them into one of two study groups, subjects were given a 2-week package of either probiotic or placebo capsules. Fasting blood samples (10 ml) before and after the intervention were collected from forearm vein by a laboratory technician for measurement of fasting blood glucose and fasting insulin at Alzahra Hospital laboratory. Plasma glucose levels were assessed using a glucose oxidase/peroxidase method as an enzymatic colorimetric (GOD-PAP) methodology [24] by Pars Azmoon test kits (Pars Azmoon Inc, Tehran, Iran). Serum insulin levels were measured by ELISA method using Monobind kit [25]. Homeostasis model assessment insulin resistance (HOMA-IR) was used to assess insulin resistance [26]. A HOMA-IR value above 3.8 is defined as insulin resistance [27]. QUICKI index (quantitative insulin sensitivity check) was used in present study to assess insulin sensitivity [28]. Details of data collection methodology and laboratory testing are published in the trial protocol elsewhere [29].

HOMA-IR was used as the main outcome for estimating the sample size. Sample size was estimated using parameters from the study by Asemi et al. assuming a maximum type 1 error of 0.05 and 90 % statistical power, HOMA-IR index standard deviation equal to 31 % and an effect size equal to 0.2; a total number of 32 subjects were estimated to be enrolled for each group taking into account 10 % attrition rate [30]. A total of 64 pregnant women with GDM were randomly allocated using block randomization techniques stratified according to the prepregnancy fasting blood sugar (FBS) and BMI groups. To ensure double blinding, a coder anonymously labelled the capsules packages as “A” or “B” and therapist assigned them according to the random sequence generated through a computer program [31]. Patients were visited in obstetrics and gynaecology clinics of Alzahra University Hospital in Tabriz. Weight and blood pressure measurements were also done every 2 weeks. Through the study course, in order to improve the compliance, the participants were contacted by telephone each week asked about any problems they may have and about gastrointestinal symptoms as well as use of any drugs through the period.

Data were analyzed using SPSS statistical software package version 22. Mean response scales measured over the 8-week study period were analyzed using appropriate statistical methods including independent samples *t* test, repeated measurements analysis of variance, one-way analysis of variance and analysis of covariance. Energy-adjusted nutrient intakes were calculated as the residuals from the regression model, with absolute nutrient intake as the dependent variable and total energy intake as the independent variable. A *p* value below 0.05 was considered as statistically significant.

## Results

**Fig. 1:**
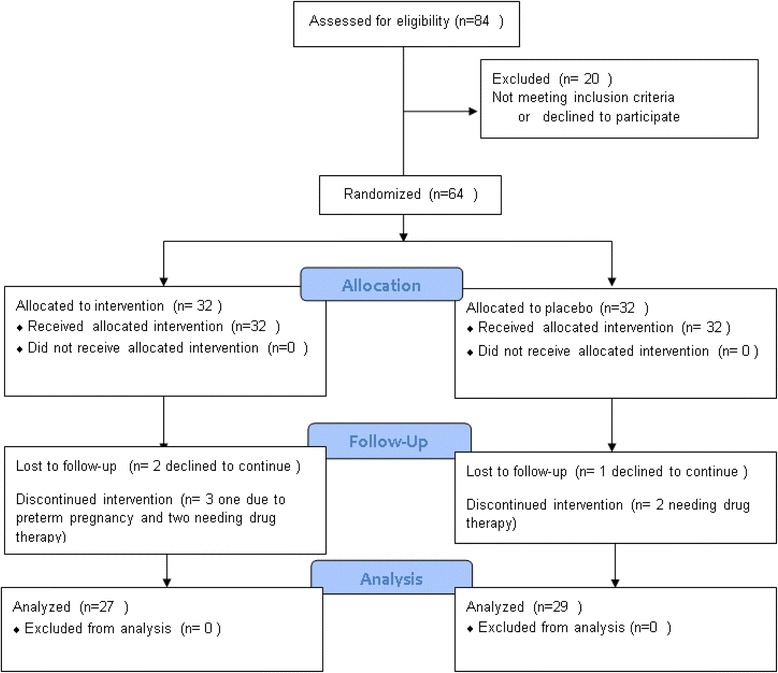
CONSORT 2010 flow diagram for the randomized placebo-controlled clinical trial for the effect of probiotics on gestational diabetes mellitus (GDM)

A total of 64 nulliparous pregnant women with gestational diabetes participated in the study, data from the 29 patients in the probiotic group and 27 in the placebo group were finally analyzed (see CONSORT flow diagram in Fig. 1). Mean age of the patients was 27.3 (SD: 5.8) years. Comparison of the baseline characteristics for both groups of pregnant women under study are presented in Table 1.

**Table 1:** Baseline comparison of demographic and anthropometric characteristics of the pregnant women with gestational diabetes mellitus enrolled either to receive probiotic or placebo

Baseline measures	Intervention	Placebo	*p* value
	(*N* = 29)	(*N* = 27)	
Maternal age	28.14 ± 6.24	26.48 ± 5.23	0.36
Family history of DM			
Yes	16 (55.2 %)	12 (44.4 %)	0.59
No	13 (44.8 %)	15 (55.6 %)	
Educational level			
Under graduate diploma	4 (13.8 %)	5 (18.5 %)	0.91
High school diploma	17 (58.6 %)	15 (55.6 %)	
Academic education	8 (27.8 %)	7 (25.9 %)	
Employment			
Employed	10 (34.5 %)	9 (33.3 %)	1.00
Unemployed or housewife	19 (65.5 %)	18 (66.7 %)	
Residence			
Urban			
Rural	17 (58.6 %)	15 (55.6 %)	1.00
	12 (41.4 %)	12 (44.4 %)	
Physical activity			
Low	22 (75.9 %)	17 (63.0 %)	
Moderate	7 (24.1 %)	10 (37.0 %)	0.38
High	0	0	
Weight (kg)	83.27 ± 12.06	78.67 ± 11.09	0.14
Height (cm)	162.68 ± 5.65	162.14 ± 5.93	0.72
Body mass index (kg/m^2^)	31.41 ± 3.92	29.86 ± 3.39	0.12

Numeric scales are reported as mean ± standard deviation, and categorical measures are reported as frequency (percent)

**Fig. 2:**
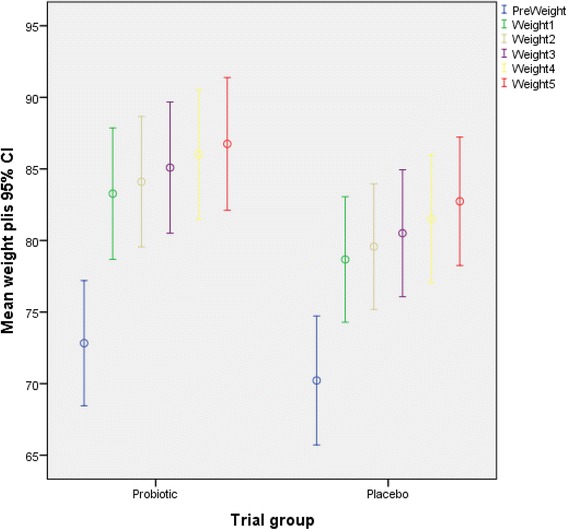
Mean weight change of women with gestational diabetes mellitus (GDM) over the 8-week study period compared between the probiotic and placebo groups

Comparing the frequency of blood glucose levels at baseline in pregnant women showed that in probiotic group, 69 % of mothers had fasting plasma sugar between 92 and 104 mg/dl and among 31 % of them, FBS ranged 105–126 mg/dl. The values in the placebo group were 66.7 and 33.3 %, respectively. There was no statistically significant difference between the groups in terms of classification of blood glucose at baseline. Mean weight change over the study period is compared between the groups in Fig. 2.

The weight gain over the study period, adjusted also for energy intake, is given in Table 2. It shows that no significant differences between the intervention and control groups were observed in weight gain measures among the pregnant women within 2-week intervals during the first 6 weeks of the study. However, in the last 2 weeks of the study, the weight gain in pregnant women in the probiotic group was significantly lower than in the placebo group. The results stayed statistically significant after adjusting for the changes in daily energy intake between the two groups, (*p* < 0.05).

**Table 2:** Weight gain trend over the 8-week study period comparing women gestational diabetes mellitus receiving probiotic supplement versus placebo capsules

Weight gain	Probiotic (*n* = 29)	Placebo (*n* = 27)	Mean difference (95 % CI)	*p* value*	*p* value**
First 2 weeks (mean ± SE)	0.83 ± 0.08	0.89 ± 0.07	−0.61 (−0.29,0.17)	0.59	0.34
Second 2 weeks (mean ± SE)	0.98 ± 0.07	0.93 ± 0.06	0.05 (−0.14,0.25)	0.60	0.69
Third 2 weeks (mean ± SE)	0.91 ± 0.06	1.00 ± 0.05	−0.09 (−0.27,0.08)	0.31	0.19
Fourth 2 weeks (mean ± SE)	0.74 ± 0.14	1.22 ± 0.11	−0.48 (−0.85,−0.10)	0.01	0.02
First 4 weeks (mean ± SE)	1.82 ± 0.12	1.82 ± 0.12	−0.008 (−0.37,0.35)	0.96	0.62
Second 4 weeks (mean ± SE)	1.65 ± 0.13	2.22 ± 0.13	−0.57 (−0.95,−0.19)	0.004	0.004

* Independent samples *t* test

**One-way ANOVA with differences in energy intake as covariate

Fasting blood sugar levels, fasting insulin level, insulin resistance index (HOMA-IR) and insulin sensitivity index (QUICKI index) were not found to be different between the probiotic supplement and placebo groups at baseline. According to the results of the analysis of covariance, FBS in both the intervention and control groups decreased significantly (14.66 and 7.38 %, respectively), both of which were statistically significant (*p* < 0.05). Post-intervention comparison, after adjustment for baseline values, showed that decrease in the probiotic group was significantly higher than in the placebo group (*p* < 0.05). Insulin resistance index in the probiotic group significantly decreased over the study period (6.74 % reduction), which was statistically significant (*p* < 0.05). In the placebo group, however, there was an increase in insulin resistance (6.45 %), but the change was not statistically significant. Insulin sensitivity index was increased in both groups. These changes in the probiotic group (5.76 %) were statistically significant. The post-intervention insulin sensitivity index in the probiotic group was not significantly different from that in the placebo group when adjusted for the baseline levels. Detailed information on changes in glucose metabolism indices are provided in Table 3.

**Table 3:** Changes in glucose metabolism indices among women with gestational diabetes receiving probiotic supplement versus placebo

Glucose metabolism indices	Probiotic (*n* = 29)	Placebo (*n* = 27)	*p* value (Independent samples *t* test)	*p* value (ANCOVA)
Fasting blood sugar (mg/dl)				
Before trial	103.65 (1.34)	100.89 (1.52)	0.17	0.02
After trial	88.37 (2.05)	93.59 (3.61)	0.20	
* P* (paired samples *t* test)	<0.001	0.02		
Absolute change	−15.27 (1.83)	−7.30 (3.04)	0.02	
Relative change	−14.66 (1.77)	−7.38 (15.09)	0.03	
Fasting serum insulin (μIU/ml)				
Before trial	5.95 (0.50)	5.60 (0.37)	0.58	0.09
After trial	5.15 (0.41)	6.12 (0.50)	0.14	
* P* (paired samples *t* test)	0.16	0.30		
Absolute difference	−0.80 (0.56)	0.52 (0.49)	0.08	
Relative difference	10.55 (18.55)	14.55 (9.21)	0.85	
HOMA-IR index				
Before trial	1.52 (0.12)	1.38 (0.08)	0.41	0.03
After trial	1.11 (0.09)	1.40 (0.11)	0.06	
* P* (paired samples *t* test)	0.007	0.93		
Absolute difference	−0.40 (0.13)	0.01 (0.12)	0.03	
Relative difference	−6.74 (14.84)	6.45 (9.22)	0.46	
QUICKI index				
Before trial	0.15 (0.00)	0.15 (0.00)	0.92	0.11
After trial	0.16 (0.00)	0.16 (0.00)	0.11	
* P* (paired samples *t* test)	0.02	0.46		
Absolute difference	0.008 (0.003)	0.002 (0.002)	0.16	
Relative difference	5.76 (2.16)	1.38 (1.69)	0.12	

*HOMA-IR* homeostasis model assessment insulin resistance, *QUICKI* quantitative insulin sensitivity check

## Discussion

It was observed in present study that the weight gain did not change during the first 6 weeks of the study, but, in the last 2 weeks of the study, the weight gain in pregnant women in the probiotic group was significantly lower than in the placebo group. With respect to glucose metabolism indices, probiotic supplementation was also effective. FBS decreased in the probiotic group significantly more than in the placebo group. Insulin resistance index in the probiotic group significantly decreased over the study period (6.74 % reduction), which was statistically significant while the increase in insulin resistance (6.45 %) in the placebo group was not statistically significant. Insulin sensitivity index was increased in both groups, but the post-intervention insulin sensitivity index in the probiotic group was not significantly different from the placebo group.

Although several studies have been done on effect of probiotics on preventing GDM and some studies have been done on general glucose patterns among normal pregnant populations, studies are scarce investigating the effect of probiotics when taken after diagnosis of GDM. One very recent double-blind randomized clinical trial on women with a new diagnosis of GDM and impaired glucose tolerance assigned them to a daily probiotic (Lactobacillus salivarius UCC118) or placebo capsule. Among the 149 women enrolled, no difference was observed between the probiotic and placebo groups in post-intervention fasting glucose levels. However, they found a likely effect for the probiotic on lipid profile of the patients [32]. Regardless of the differences in study design and population, the main characteristic possibly describing the reason for positive results in present study may be attributed to different probiotic contents of the supplement in two studies. Observing the effect of probiotics in present study with a smaller sample size while yielding reasonably narrower confidence interval justifies a promising effect for the intervention used in our study. Aside from the different probiotic in present study and the magnitude of potential random error attributable to the sample size, other issues may affect the results either such as the variations in normal weight gain through the pregnancy, variations in gut flora, physical activity and diet. Normally, such variations are controlled through randomization, but this cannot be fully guaranteed unless the study is large enough to ensure the efficacy of randomization [33]. No doubt physical activity is a major risk indicator in several noncommunicable diseases including diabetes. In present study, the physical activity was measured only at baseline, and we recommend to consider it as a time-dependent variable in future clinical trial studies assessing the role of probiotics on GDM. Studies like present study conducted specifically on patients diagnosed with GDM are quite rare; however, there are bunches of studies on role of probiotics on prevention of GDM or its related pregnancy outcomes. Generally, the effect of probiotics in prevention of GDM and poor pregnancy outcomes have been shown to be promising [6, 20, 21, 34–39]. Probiotics have also been investigated when combined with diet recommendations. In a study from Finland, 256 pregnant women were randomized early through the gestation to receive nutrition counselling or not to receive it as controls; the group with dietary intervention was further randomized to receive probiotics (Lactobacillus rhamnosus GG and Bifidobacterium lactis Bb12; diet/probiotics) or placebo (diet/placebo). It was found that blood glucose levels as well as HOMA-IR and QUICKI indices were lowest in the diet/probiotics group concluding that improved blood glucose control could be reached by dietary counselling combined with probiotics even in normoglycaemic pregnant women [40]. A recent review on dietary interventions, lifestyle changes and dietary supplements in preventing GDM has concluded that trials in which only intake or expenditure has been the target of interventions may not achieve positive efficacy results, whereas combined interventions and dietary and lifestyle interventions could show better efficacy in the reduction of GDM prevalence. The same review has stated the results of probiotic studies to be promising and has recommended future larger scale studies [41]. Although, the cost-effectiveness of probiotics has not been well documented in literature, probiotic supplements have been shown to have acceptability among patients [42, 43].

About 95 % of the intestinal microbiome in healthy people with normal weight consists of the species belonging to three phyla of the bacteria including *Bacteroidetes*, *Firmicutes* and *Actinobacteria*. The plausibility of the observed effect of probiotics is not much hard to be approved considering the available evidence on role of healthy gut microbiome on carbohydrate metabolism and the synergistic relationships of probiotic foods and supplements with the host gut microbiome [21, 44, 45]. To complete the plausibility pattern, the changes in gut microbiome during diabetes mellitus should also be considered. A new theory suggests that gut microbiota have a role in the regulation of the energy homeostasis and causing metabolic diseases and insulin resistance [46]. It has been shown that type 2 diabetes is associated with changes in gut microbiome. For instance, it has been shown that the ratio of *Bacteroidetes* to *Firmicutes* species is associated with diabetes type 2 and FBS, especially that the changes in gut microbiome are different from what is observed during obesity and a larger number of opportunistic pathogens along with a lower number of butyrate-producing bacteria are attributed to presence of diabetes [21, 47–51].

## Conclusions

The probiotic supplement appeared to affect glucose metabolism and weight gain among pregnant women with GDM.

### Ethical issues

Study protocol was approved by the Research Ethics committee of International branch of Shahid Beheshti University of Medical Sciences as a thesis proposal for PhD degree in Nutrition Sciences. Written informed consent was taken from all participants.

The clinical trial was registered in the Iranian Registry of Clinical Trials with reference number IRCT201405181597N3, available at: http://www.irct.ir, accessible through world health organization database of clinical trial registries.

### Statement of human and animal rights

All procedures followed were in accordance with the ethical standards of the responsible committee on human experimentation (institutional and national) and with the Helsinki Declaration of 1975, as revised in 2008 (5).
